# The Beneficial Effects of Physical Activity: Is It Down to Your Genes? A Systematic Review and Meta-Analysis of Twin and Family Studies

**DOI:** 10.1186/s40798-016-0073-9

**Published:** 2017-01-10

**Authors:** J. R. Zadro, D. Shirley, T. B. Andrade, K. J Scurrah, A. Bauman, P. H. Ferreira

**Affiliations:** 1 Discipline of Physiotherapy, Faculty of Health Sciences, The University of Sydney, 75 East Street, Lidcombe, Sydney, NSW 1825 Australia; 2 Australian Centre for Excellence in Twin Research, Centre for Epidemiology and Biostatistics, Melbourne School of Population and Global Health, The University of Melbourne, Melbourne, Australia; 3 School of Public Health and Charles Perkins Centre, University of Sydney, Sydney, Australia

**Keywords:** Genetics, Heritability, Familial aggregation, Physical activity, Body composition, Cardiorespiratory fitness

## Abstract

**Background:**

There is evidence for considerable heterogeneity in the responsiveness to regular physical activity (PA) which might reflect the influence of genetic factors. The aim of this systematic review was to assess whether the response to a PA intervention for measures of body composition and cardiorespiratory fitness is (i) correlated within twin pairs and/or families and (ii) more correlated in monozygotic twins (MZ) compared to dizygotic twins (DZ), which would be consistent with genetic effects.

**Methods:**

We performed electronic database searches, combining key words relating to “physical activity” and “genetics”, in MEDLINE, CINAHL, EMBASE, SPORTS Discuss, AMED, PsycINFO, WEB OF SCIENCE, and SCOPUS from the earliest records to March 2016.

Twin and family studies were included if they assessed body composition and/or cardiorespiratory fitness following a PA intervention, and provided a heritability estimate, maximal heritability estimate, or within MZ twin pair correlation (r_MZ_).

Data on heritability (twin studies), maximal heritability (family studies), and the r_MZ_ were extracted from included studies, although heritability estimates were not reported as small sample sizes made them uninformative.

**Results:**

After screening 224 full texts, nine twin and five family studies were included in this review. The pooled r_MZ_ in response to PA was significant for body mass index (r_MZ_ = 0.69, *n* = 58), fat mass (r_MZ_ = 0.58, *n* = 48), body fat percentage (r_MZ_ = 0.55, *n* = 72), waist circumference (r_MZ_ = 0.50, *n* = 27), and VO_2_max (r_MZ_ = 0.39, *n* = 48), where “*n*” represents the total number of twin pairs from all studies. Maximal heritability estimates ranged from 0–21% for measures of body composition, and 22–57% for cardiorespiratory fitness.

Twin studies differed in sample age, baseline values, and PA intervention, although the exclusion of any one study did not affect the results.

**Conclusions:**

Shared familial factors, including genetics, are likely to be a significant contributor to the response of body composition and cardiorespiratory fitness following PA.

Genetic factors may explain individual variation in the response to PA.

**Trial Registrations:**

PROSPERO Registration No CRD42015020056.

**Electronic supplementary material:**

The online version of this article (doi:10.1186/s40798-016-0073-9) contains supplementary material, which is available to authorized users.

## Key points

- Shared familial factors, including genetics, are likely to play a stronger role in the response of body composition when compared to cardiorespiratory fitness.
- The response of body mass index, fat mass, and body fat percentage to PA appear to be more dependent on shared familial factors than measures such as waist-to-hip ratio.
- These results have implications for the management of conditions which advocate increased levels of PA, since shared familial factors, including genetics, might serve as an explanation for why some people respond more effectively than others in specific measures of PA.

## Background

Engagement in regular physical activity (PA) is one of the most important aspects for maintaining optimal health and is recommended for reducing the risk of numerous diseases (including cardiovascular disease) in people of all ages [1–4]. In addition, PA is used as a non-pharmacological treatment option for coronary heart disease [5], osteoporosis [6], rheumatoid arthritis [7], anxiety disorders [8], and a variety of musculoskeletal conditions, including low back pain [9]. Although the benefits of PA are numerous, their positive effects on cardiorespiratory fitness [e.g., maximal oxygen uptake (VO_2_max)] and measures of body composition [e.g., body mass index (BMI)] [10] deserve special attention, due to their subsequent influence on cardiovascular disease and mortality rates. Cardiorespiratory fitness is a strong and independent risk factor for cardiovascular disease and all-cause mortality [11], with up to 7% of deaths being attributed to low cardiorespiratory fitness [12]. Similarly, high values of body composition measures, such as BMI and waist circumference, are significantly associated with greater all-cause [13] and CVD-related mortality [14].

Although the benefits of PA are clear and substantial, research has demonstrated that genetic factors have a strong influence on PA engagement [15], with the heritability of time-spent in moderate-to-vigorous intensity PA estimated at 47% [16]. In addition, not everyone engaged in PA will benefit to the same extent, with strong evidence for considerable heterogeneity in the responsiveness to regular PA [17–19]. This variation might also reflect the influence of genetic factors.

Twin and family studies are commonly used to investigate the extent to which shared familial factors, including genetics, contribute to the variation of a phenotype. Monozygotic (MZ) twins share 100% of their segregating genes, while dizygotic (DZ) twins share 50% on average. If genes influence a phenotype, we would expect to see a greater correlation for MZ twins than for DZ twins, and if genes are the only influence on a phenotype the ratio should be 2:1, with a heritability estimate of 100%. Smaller differences between the correlations would indicate that shared environmental effects are involved, with the shared environment referring to the exposure to similar environmental (non-genetic) factors within twin pairs (e.g., nutrition, physical activity, childhood experiences, parental beliefs and values, socioeconomic status, etc.). Family studies can estimate maximal heritability using correlations between parent-offspring pairs and siblings (sometimes adjusted for correlation between spouses) [19]. However, unlike heritability estimates from twin studies, these studies are unable to tease apart the contribution from genetic and shared environmental factors. This is because different proportions of genetic sharing are required to separate genetic and shared environmental sources of variation, and in nuclear families parent-offspring pairs and sibling-pairs share equal proportions of their genes (50%). Although we can estimate spouse correlations, we cannot tell whether this correlation is due to shared genes (assortative mating) or shared environmental factors.

The role of both genetic and environmental factors shared within families in the response to a PA intervention has been investigated in a number of studies. MZ twin pairs who completed a standardized PA intervention demonstrated great variation in the amount of weight lost between twin pairs, but only a small amount of variation within twin pairs [20]. In addition, individual differences in the response of VO_2_max following an exercise program were 2.5 times more variable between families than within families [19]. These results suggest that factors shared within families, including genes, play a role in the response to a PA intervention, although their exact contribution, across measures of body composition and cardiorespiratory fitness, are not well understood. A better understanding of the contribution genetics and shared environmental effects make to people’s response to PA may help health practitioners understand the possible reasons behind individual variation in response to a PA targeted intervention, and why some patients demonstrate a more favorable response.

The aim of this systematic review is to obtain quantitative estimates of twin correlations (both MZ and DZ), heritability (from twin studies), and maximal heritability (from family studies), for measures of body composition and cardiorespiratory fitness in response to a PA intervention.

## Methods

### Search Strategy

We conducted a systematic review and meta-analysis in accordance with the “Preferred reporting items for systematic reviews and meta-analyses” (PRISMA) statement [21]. The protocol for this systematic review has been registered on PROSPERO (Registration No: CRD42015020056). We performed electronic database searches in MEDLINE, CINAHL, EMBASE, SPORTS Discuss, AMED, PsycINFO, WEB OF SCIENCE, and SCOPUS from the earliest records to May 2015. The search was then updated in March 2016. We used a comprehensive key word search strategy (Additional file 1) combining key words relating to PA (e.g., “physical activi*” OR “exercise” OR “resistance training” etc.) and genetics (e.g., “genetic*” OR “herita*” OR “family resemblance” etc.). The search strategy remained sensitive to capture all outcomes related to body composition and cardiorespiratory fitness. To identify additional studies we performed a hand search of the reference lists from included papers.

### Study Selection

Two reviewers (TA and JZ) independently performed the selection of studies and consensus was used to resolve any disagreement. Studies were included if they investigated clinically relevant outcome measures of body composition or cardiorespiratory fitness following a PA or exercise intervention (referred to hereafter as PA interventions) amongst twin pairs and/or family members. Studies investigating a PA intervention in combination with other interventions (e.g., diet) were included. We included randomised controlled trials and case series provided they reported a within MZ twin pair correlation (r_MZ_), heritability estimate (from a twin study), or maximal heritability estimate (from a family study). Heritability estimates and the r_MZ_ for the response of an intervention (based on change scores) are commonly reported in studies where twin pairs are considered as clusters, with the treatment effect as a fixed variable [22]. To investigate the intra-pair resemblance in the response to PA it is essential that twin pairs participate in an identical intervention. This is similar to the methodology employed in family studies to obtain a maximal heritability estimate (where the variance explained by genetic and shared environmental factors cannot be teased apart). Therefore, we decided not to use methodological quality as part of the inclusion/exclusion criteria as it is not practical to consider items commonly assessed in systematic reviews of randomized controlled trials (such as allocation concealment, blinding, and intention-to-treat) [23] when considering this study design. It is unlikely results from twin and family studies investigating heritability are subject to publication bias, since the contribution of genetics and shared environment is relevant regardless of whether the estimates are small or large. However, we acknowledge the possibility that individual studies may only report results for traits that demonstrate a high heritability. To minimize the risk of reporting bias, we contacted authors when there was data available on body composition and cardiorespiratory fitness but within twin pair correlations were not reported. Observational studies or studies only assessing the heritability of PA engagement, without a PA intervention, were excluded. There was no restriction on the age or gender of participants, nor the type of PA intervention investigated. We included published conference abstracts and dissertations provided they met the inclusion criteria.

### Data Extraction

Two reviewers (DS and JZ) independently performed the extraction of data. A standardized data extraction form was used to collect data on participants’ characteristics (age, gender, and zygosity), sample size, prescribed PA intervention (frequency, intensity, duration, and type), co-prescription of other interventions (e.g., diet), outcomes assessed, loss to follow up, and study type.

### Data Analysis

Data on correlation (*r*), equality of variances (*F*), heritability (*h* ^2^), and maximal heritability were extracted from included studies. In family studies, “heritability” estimates were derived from the familial correlation model and termed “maximal heritability”, since the model is unable to partition the variance explained by genetic and non-genetic sources shared within families [24]. In twin studies, heritability estimates were calculated from the following formula: *h* ^2^ = 2(r_MZ_–r_DZ_), where r_DZ_ is the within DZ twin pair correlation. When *h* ^2^ was greater than 1 we used r_MZ_ as the heritability estimate, since it is not possible for genetics to contribute more than 100% to the variance of a phenotype. In addition, if there were no data available for DZ twins, we used r_MZ_ as an estimate of the upper bound of heritability (including variance from genetic and shared environmental factors). In cases where the F-ratio was reported but the r_MZ_ was not, we used the following formula to calculate r_MZ_ as described by Haggard: *r* = (F–1)/(*F* + 1) [25]. Authors were contacted when required data were not published. When raw data were obtained from twin studies, we attempted to fit variance components models to change scores in order to estimate the r_MZ_ and r_DZ_ simultaneously and formally compare models in which these two parameters were forced to be equal with models in which they were allowed to differ. However, for many phenotypes the models could not be fitted or failed to converge due to small sample sizes (no results shown from these models). Instead, for all phenotypes, and separately for MZ and DZ twin pairs, we performed a one-way (twin pair identifier) analysis of variance with change score as the outcome (calculated from the pre and post-intervention raw data). Specifying change score as a repeated measure within a twin pair in the models enabled calculation of the within twin pair correlation. When possible and applicable, we adjusted the analyses for age, gender, and baseline values [22]. If studies were considered homogenous in terms of outcomes and PA interventions, we performed a meta-analysis using Comprehensive Meta-Analysis Version 3.0. Additionally, if there were enough studies investigating PA interventions of varying durations, the co-prescription of other interventions (e.g., diet), or analyzing data from males and females separately, we sub-grouped our meta-analyses accordingly. If pooling data on heritability/maximal heritability was not possible (from either twin or family studies), we attempted to pool data on the r_MZ_. Data on correlation and sample size from each study with greater than or equal to four twin pairs (the minimum number of observations allowed to be entered into the software) was used to provide a pooled estimate of the r_MZ_, 95% confidence interval (CI), and p-value. Heterogeneity between studies was assessed using the *I* ^2^ statistic. An *I* ^2^ value <25% indicates low heterogeneity between studies. We used fixed-effects where *I* ^2^ was <50% and random-effects when *I* ^2^ was ≥50% (moderate heterogeneity). We did not display pooled estimates where the *I* ^2^ value indicated high heterogeneity (≥75%) [26].

## Results

### Description of Studies

**Fig. 1:**
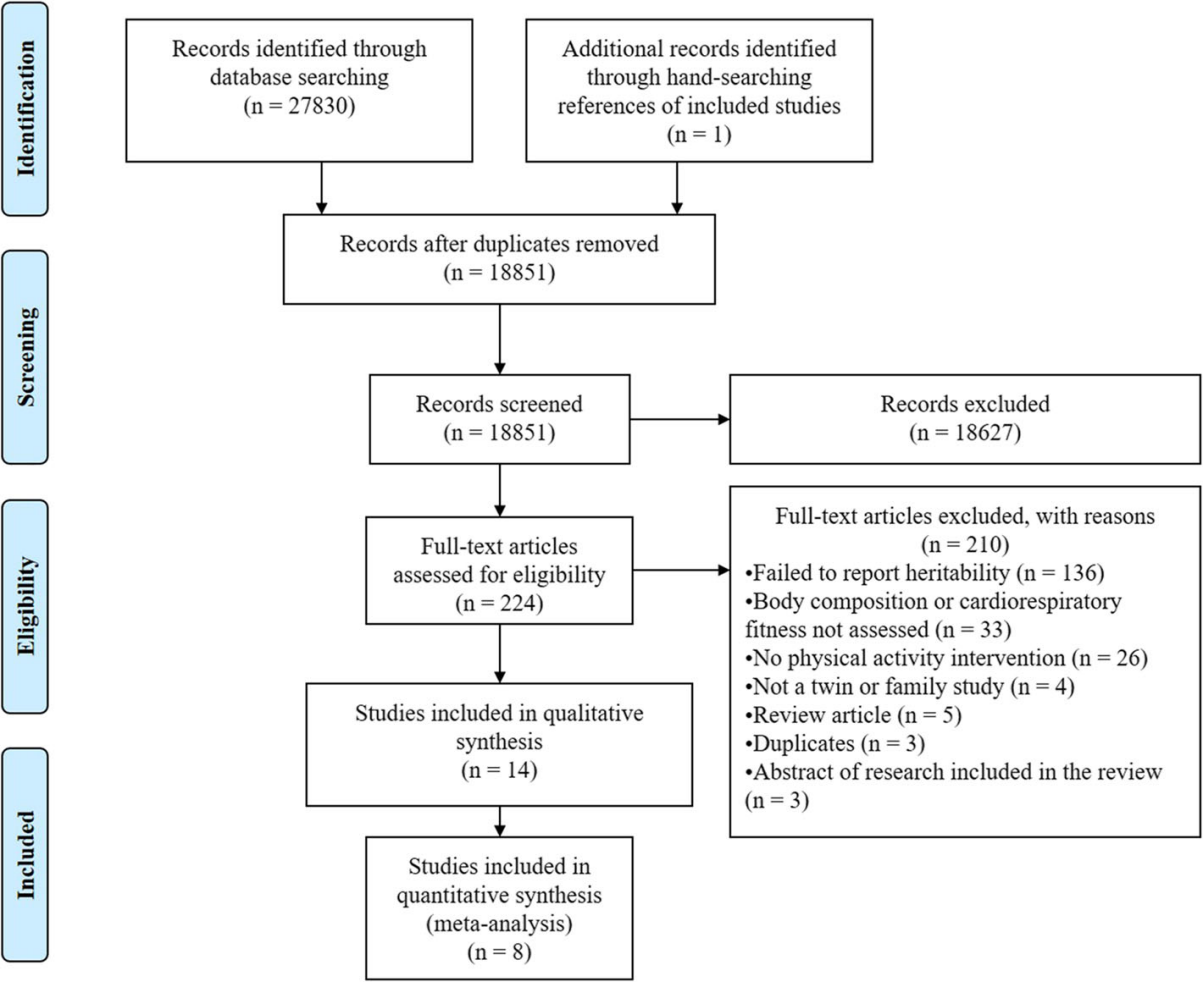
PRISMA flow diagram

The comprehensive key word search yielded 27,830 results, with one additional study retrieved from hand searching the reference lists of included studies. After removing duplicates and screening titles and abstracts there were 224 full texts which were screened. A total of 14 studies (nine twin and five family studies) were included in this systematic review, with eight twin studies forming the basis for our meta-analyses (Fig. 1). The nine twin studies included data from a total of 83 complete MZ twin pairs, and 15 complete DZ twin pairs, with no twin pairs used in more than one study (as confirmed by authors named in multiple included studies). The five family studies were based off the same sample of 199 families (which did not include any twin pairs). Although there were numerous twin and family studies similar in design and outcomes, we were unable to pool heritability estimates for any outcomes for two main reasons. First, there were an insufficient number of family studies deriving results from independent samples. Second, although we were able to obtain heritability estimates from three twin studies, these estimates were uninformative since the 95% CI covered the whole range (0,1) (apart from Danis and colleagues who estimated heritability without utilizing DZ twins in its design [27]), and differences between the r_MZ_ and r_DZ_ were not statistically significant (Table 1).Instead, we were able to pool the r_MZ_ for selected outcomes, giving us quantitative estimates of the upper bound of heritability. Included studies that reported more than one outcome measure were used in multiple meta-analyses.

**Table 1:** Within MZ and DZ twin pair correlations for the response of body composition and cardiorespiratory fitness following a physical activity intervention in twin studies

Author (year)	Sample	Age [mean (SD)]	Baseline status [mean (SD)]	Within MZ correlation (95% CI)	Within DZ correlation (95% CI)	Between MZ and DZ correlation significance^d^
Body fat percentage (%)						
Hopkins ND (2012)^a^	6 MZ (1 male and 5 female) and 6 DZ (2 male and 4 female) twin pairs	MZ: 13.5 (0.8) DZ: 13.4 (0.8)	MZ: 27.1 (6.9) DZ: 26.0 (11.3)	0.63 (−0.37 to 0.95)	0.31 (−0.67 to 0.90)	*p* = 0.606
Afman G (1988)^b^	18 MZ (2 males and 16 females) and 9 DZ (3 males and 6 females) twin pairs	MZ: 19.0 (1.4) DZ: 19.4 (1.8)	MZ: 21.3 (9.0) DZ: 19.9 (7.2)	0.61 (0.20 to 0.84)	0.50 (−0.25 to 0.87)	*p* = 0.742
Danis A (2003)	9 MZ male twin pairs	11–14^c^	E: 17.8 (4.1) C: 16.8 (2.8)	*	*	*h* ^2^ = 69%**
BMI						
Hopkins ND (2012)^a^	6 MZ (1 male and 5 female) and 6 DZ (2 male and 4 female) twin pairs	MZ: 13.5 (0.8) DZ: 13.4 (0.8)	MZ: 21.5 (3.5) DZ: 21.9 (3.5)	0.81 (0.00 to 0.98)	0.57 (−0.45 to 0.94)	*p* = 0.557
Afman G (1988)^b^	16 MZ (3 males and 13 females) and 6 DZ (2 males and 4 females) twin pairs	MZ:18.6 (1.1) DZ: 19.3 (1.3)	MZ: 21.9 (1.9) DZ: 22.6 (3.7)	0.42 (−0.10 to 0.76)	0.00 (−0.81 to 0.81)	*p* = 0.485
Weight (kg)						
Hopkins ND (2012)^a^	6 MZ (1 male and 5 female) and 6 DZ (2 male and 4 female) twin pairs	MZ: 13.5 (0.8) DZ: 13.4 (0.8)	MZ: 59.0 (11.5) DZ: 58.9 (12.6)	0.89 (0.28 to 0.99)	0.00 (−0.81 to 0.81)	*p* = 0.091
Afman G (1988)^b^	19 MZ (3 males and 16 females) and 9 DZ (3 males and 6 females) twin pairs	MZ: 18.7 (1.0) DZ: 19.4 (1.8)	MZ: 60.4 (10.6) DZ: 67.1 (13.4)	0.53 (0.10 to 0.79)	0.13 (−0.58 to 0.73)	*p* = 0.337
Fat free mass						
Hopkins ND (2012)^a^	6 MZ (1 male and 5 female) and 6 DZ (2 male and 4 female) twin pairs	MZ: 13.5 (0.8) DZ: 13.4 (0.8)	MZ: 69.9 (6.8)% DZ: 69.9 (6.8)%	0.52 (−0.50 to 0.94)	0.34 (−0.65 to 0.90)	*p* = 0.785
Afman G (1988)^b^	19 MZ (3 males and 16 females) and 9 DZ (3 males and 6 females) twin pairs	MZ: 18.9 (1.4) DZ: 19.4 (1.8)	MZ: 48.2 (8.1) kg DZ: 53.2 (13.2) kg	0.40 (−0.07 to 0.72)	0.18 (−0.55 to 0.75)	*p* = 1.000
Relative VO_2_ max (mL.kg^−1^min^−1^)						
Hopkins ND (2012)^a^	6 MZ (1 male and 5 female) and 6 DZ (2 male and 4 female) twin pairs	MZ: 13.5 (0.8) DZ: 13.4 (0.8)	MZ: 44.4 (8.1) DZ: 45.7 (8.1)	0.43 (−0.59 to 0.92)	0.21 (−0.73 to 0.87)	*p* = 0.763
Afman G (1988)^b^	19 MZ (3 males and 16 females) and 9 DZ (3 males and 6 females) twin pairs	MZ: 18.9 (1.4) DZ: 19.4 (1.8)	MZ: 33.3 (7.3) DZ: 37.1 (8.0)	0.44 (0.00 to 0.74)	0.00 (−0.66 to 0.66)	*p* = 0.324
Danis A (2003)	9 MZ male twin pairs	11–14^c^	E: 52.1 (3.6) C: 54.0 (3.9)	*	*	h^2^ = 44%**
Absolute VO_2_ max (L.min^−1^)						
Afman G (1988)^b^	20 MZ (3 males and 16 females) and 9 DZ (3 males and 6 females) twin pairs	MZ: 18.9 (1.4) DZ: 19.4 (1.8)	MZ: 2.0 (0.6) DZ: 2.5 (0.9)	0.44 (0.00 to 0.74)	0.00 (−0.66 to 0.66)	*p* = 0.320
Danis A (2003)	9 MZ male twin pairs	11–14^c^	E: 2.1 (0.4) C: 2.1 (0.4)	*	*	*h* ^2^ = 54%**

*MZ* monozygotic, *DZ* dizygotic, *E* experimental group, *C* control group, *SD* standard deviation, *CI* confidence interval, *h* ^*2*^ heritability, *VO*_*2*_ *max* maximal oxygen uptake, *BMI* body mass index

*No reported correlation due to a different method used to estimate heritability

**Unable to calculate the standard error and thus present the 95% CI

^a^Within twin pair correlations (95% CI) extracted from the publication

^b^Within twin pair correlations (95% CI) calculated from raw data

^c^Did not report a mean age (SD)

^d^Unable to calculate the within MZ and DZ twin pair correlations for Danis A (2003) due to methodology, so the *h* ^2^ is presented instead

The characteristics of the included twin and family studies, including sample size, age, baseline PA status, and PA intervention are described in Tables 2 and 3. The mean age [standard deviation (SD)] of participants ranged from 13 (1) to 39 (2) in twin studies, and 17 to 65 years in family studies. At study entry, participants were mostly sedentary or engaged in light PA but not highly physically trained. Only two twin studies [20, 28] analysed data from twin pairs living apart at the time of enrolment [mean age (SD) 30 (8) and 39 (2), respectively], while another reported that more than 50% of the twin pairs were living together at this time [mean age (SD) 19 (2)] [29]. Every study recruited healthy individuals from the community, except Hainer and colleagues [28] who recruited twin pairs admitted to an obesity unit for a 40-day PA and diet program. The frequency of the PA interventions ranged from three times a week to daily, with the duration ranging from 15 min to 2 h. The exercise intensity ranged from 50 to 97% VO_2_max, with numerous modes of PA being utilized, including a cycle ergometer, resistance training, walking or running, over a period of 22 days up to 6 months. Two twin studies [29, 30] reported drop outs based on participants failing to complete the training protocol (Table 2), while the included family studies only analyzed data from participants who completed 60 exercise sessions in 21 weeks [31] (Table 3).

**Table 2:** Characteristics of twin studies

Twin studies					
Author (year)	Sample^*^	Age [mean (SD)]	Baseline physical activity status	Physical activity intervention	Diet intervention
Poehlam A (1987)	6 MZ male twin pairs	19 (1.3)	Sedentary	F: 22 consecutive days I: 56% VO2 max T: 116 min per day T: Cycle ergometer	Energy balance deficit of ~4.2 MJ/day
Koenigstorfer J (2011)	6 MZ females twin pairs	30 (8)	Sedentary	F: 3 times per week (aerobic) and 2 times per week (strength) for 8 weeks I: 68% (±8%) heart rate maximum (aerobic) and 70% of 12 repetition maximum (12RM) T: 45 min each T: Cycle ergometer and strength training (crunches, butterfly crunches, leg press, leg curl, and latissimus pull down)	Individual counseling for a low fat (25%), hypocaloric diet (5.0–5.8 MJ/day) in accordance with their usual eating patterns and preferences
Hopkins ND (2012)	6 MZ (1 male and 5 female) and 6 DZ (2 male and 4 female) twin pairs	MZ: 13.5 (0.8) DZ: 13.4 (0.8)	Light and moderate physical activity	F: 3 times per week for 8 weeks I: 65–85% heart rate maximum T: 45 min T: gym-based aerobic exercise	None
Bouchard C (1994)	7 MZ male twin pairs^a^	21.0 (2.7)	Sedentary	F: Twice per day every 9 of 10 days for 93 days I: 50–55%VO_2_ max T: 60 min T: Cycle ergometer	Energy balance deficit of ~4.2 MJ/day
Hainer V (2000)	14 MZ female twin pairs	39 (1.7)	Sedentary	F: Daily for 28 days I: 60%VO2 max T: 20 min T: cycle ergometer aerobic exercises Additional exercise: 4 km walk and 30 min of aerobic exercise	Hypocaloric diet of 1.6 MJ/day
Hamel P (1986)	6 MZ twin pairs (3 male and 3 female)	21.2 (3.7)	Not reported	F: 3–5 times per week for 15 weeks I: 60–85% heart rate reserve T: 30–45 min T: Cycle ergometer	None
Prud’Homme D (1984)	10 MZ twin pairs (4 male and 6 female)	20.0 (2.9)	None highly trained but some participated in recreational activities	F: 4–5 times per week for 20 weeks I: 60–85% heart rate reserve T: 40–45 min T: Cycle ergometer	None
Afman G (1988)	19 MZ (3 male and 16 female) and 9 DZ (3 male and 6 female) twin pairs^b^	MZ: 18.9 (1.4) DZ: 19.4 (1.8)	Not reported	F: 4 times per week for 11 weeks I: 70–85 heart rate maximum T: 15–45 min T: cycle ergometer and treadmill running	None
Danis A (2003)	9 MZ male twin pairs	11–14**	Not participating in sporting activities	F: 3 times per week for 6 months I: 75–97% VO2 max T: 60–90 min T: treadmill running	None

*MZ* monozygotic, *DZ* dizygotic, *MJ* mega joules, *SD* standard deviation, *FITT* frequency, intensity, time, type

*Twin pairs were generally living together at the time of enrollment, except those in Koenigstorfer J [20] and Hainer V (2000) [28]. Afman G [29] reported that more than 50% of the twin pairs were living together at the time of enrollment

**Did not report a mean age (SD)

^a^11 MZ twin pairs were initially enrolled but only seven MZ twin pairs completed the exercise protocol (the definition of ‘completing the exercise protocol’ was not outlined)

^b^34 twin pairs (MZ and DZ) were initially enrolled but only 28 twin pairs (MZ and DZ) completed the protocol (defined as attending 75% or more of the exercise sessions, and having fewer than eight sessions where one twin participated and the co-twin did not)

**Table 3:** Characteristics of family studies

Family Studies (all studies were based on the sample from “The HERITAGE Family Study”)					
Author (year)	Sample^a^	Age	Baseline physical activity status	Physical activity intervention	Diet intervention
Rice T (1999)	98 Caucasian families (440 individuals)	Parents were less than 65 years old, while offspring ranged from 17–40 years old	Sedentary	F: 3 times per week for 20 weeks I: 55–75% VO_2_ max T: 30–50 min T: Cycle ergometer	None.
Bouchard C (1999)	98 Caucasian families (481 individuals)				
Perusse L (2000)	99 Caucasian families (483 individuals)				
Perusse L (2001)	99 Caucasian families (483 individuals)				
Gaskill SE (2001)	100 Caucasian families (339 individuals) and 99 African-American families (172 individuals)				

*FITT* frequency, intensity, time, type, *VO*_*2*_ *max* maximal oxygen uptake

^a^Participants needed to complete 60 exercise sessions within 21 weeks to satisfy the protocol and be included in the study

Due to significant between-study variation for the intervention frequency and duration, we were unable to stratify meta-analyses in this way. Instead, we examined the correlations for each outcome to investigate if studies with more frequent bouts of PA, or longer intervention durations reported higher r_MZ_, but, we were unable to identify any trends. We were able to stratify our meta-analyses by the co-prescription of a diet intervention, and by gender.

### Outcomes of Body Composition

**Fig. 2:**
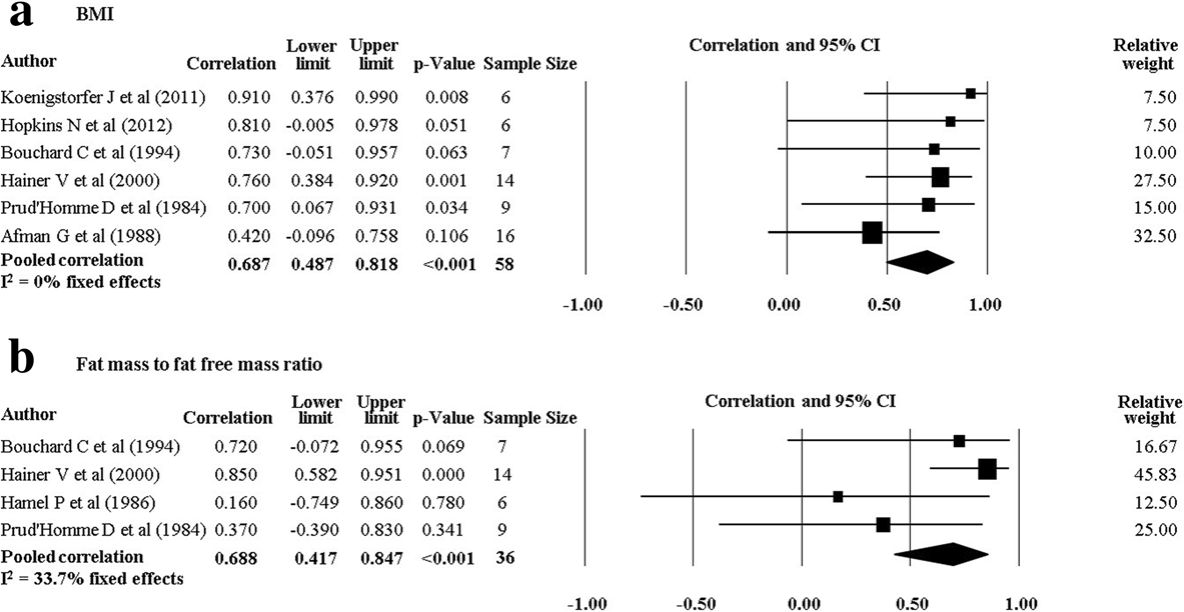
Pooled within monozygotic (MZ) twin pair correlations for BMI and the ratio of fat mass to fat free mass in response to physical activity. CI: confidence interval; sample size; number of twin pairs

**Fig. 3:**
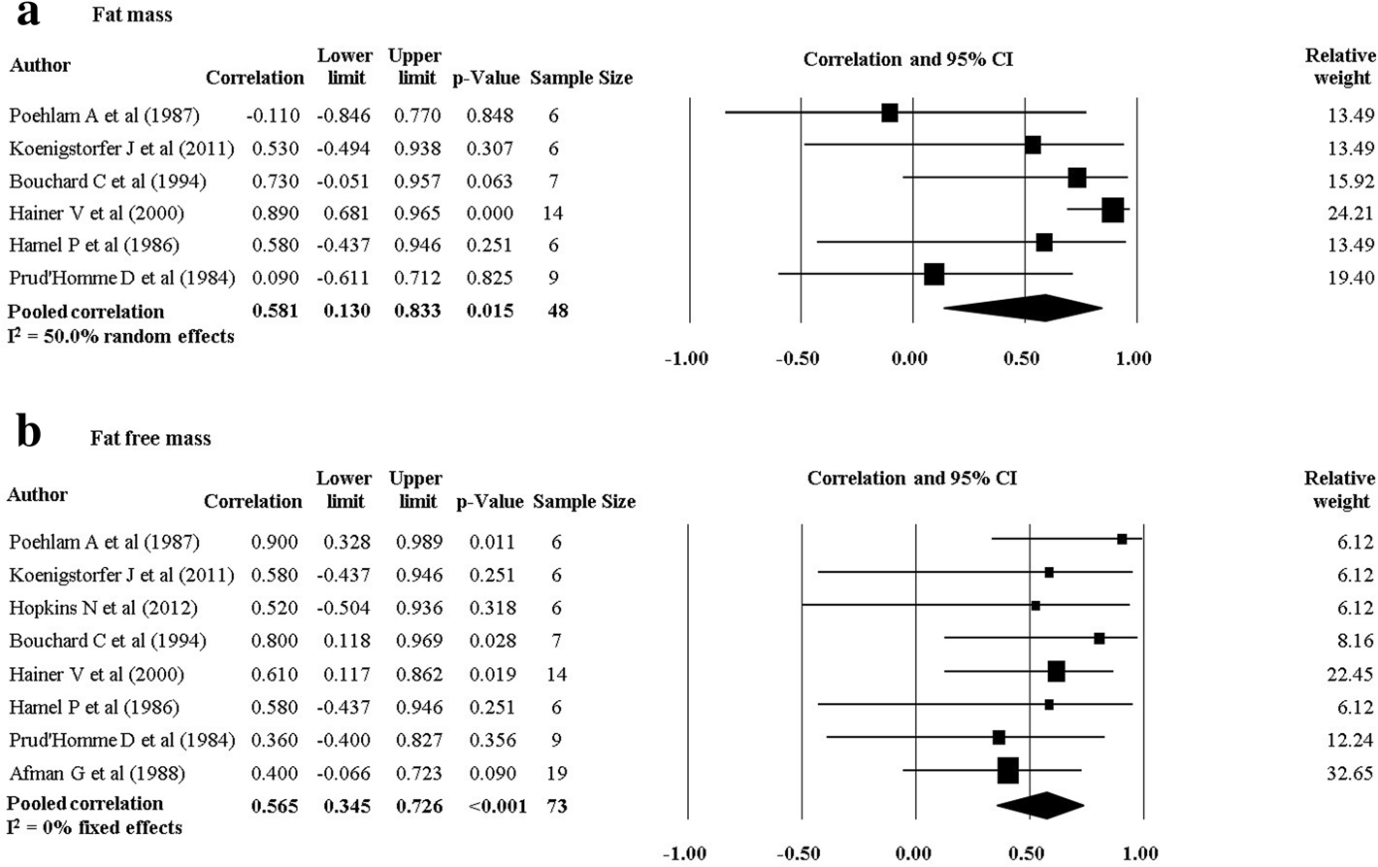
Pooled within monozygotic (MZ) twin pair correlations for fat mass and fat free mass in response to physical activity. CI: confidence interval; sample size; number of twin pairs

**Fig. 4:**
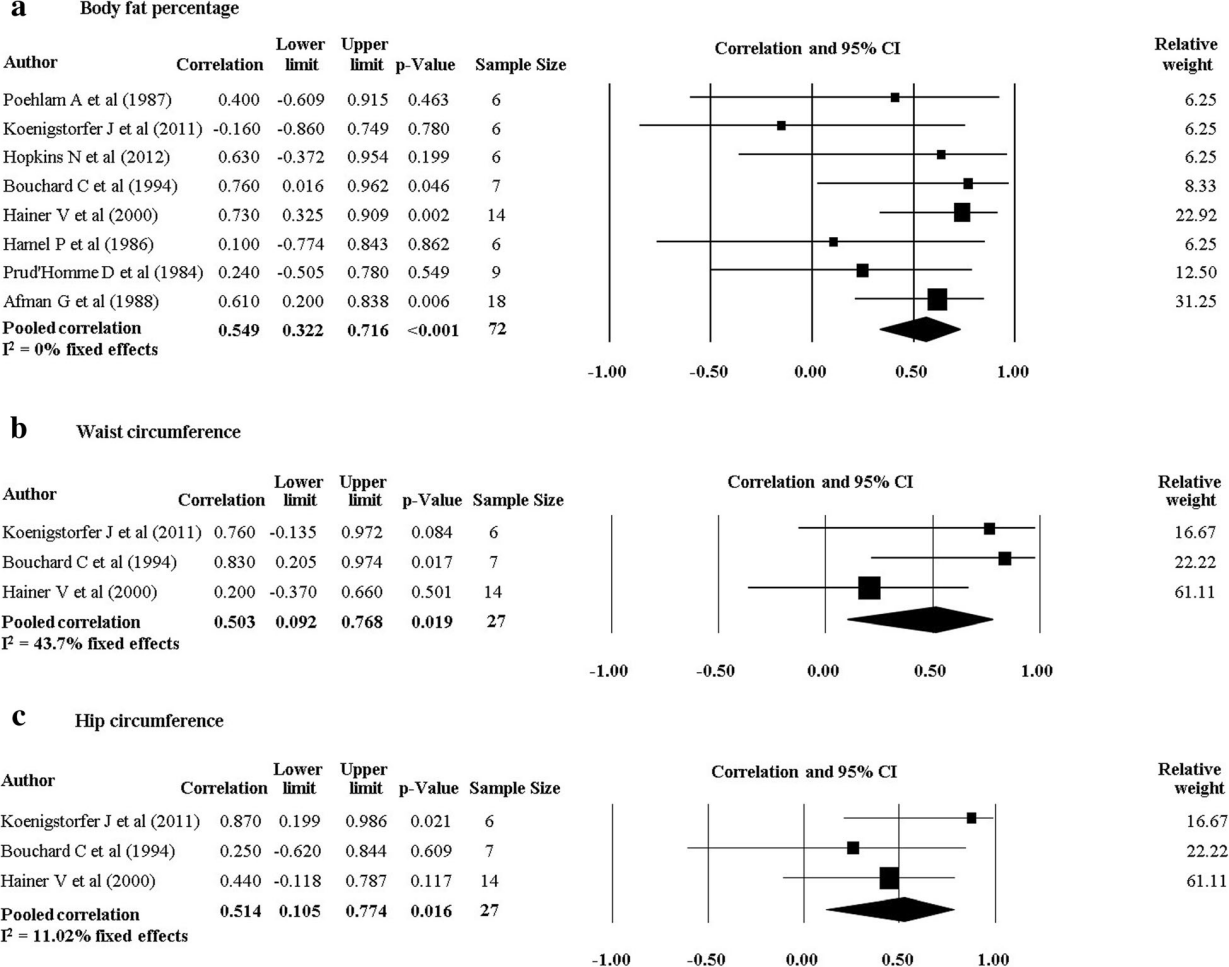
Pooled within monozygotic (MZ) twin pair correlations for body fat percentage, waist circumference and hip circumference in response to physical activity. CI: confidence interval; sample size; number of twin pairs

**Fig. 5:**
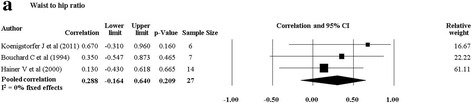
Pooled within monozygotic (MZ) twin pair correlations for waist-to-hip ratio in response to physical activity. CI: confidence interval; sample size; number of twin pairs

There were 11 studies (nine twin studies [20, 27–30, 32–35] and two family studies [24, 36]) which investigated body composition measures and their response following a PA intervention. Pooling of eight twin studies results (excluding Danis and colleagues [27] due to different methodology) suggest there is a significant r_MZ_ across the majority of body composition measures (Table 4). The pooled r_MZ_ was highest for BMI (r_MZ_ = 0.69, 95% CI: 0.49–0.82, *n* = 58) and the ratio of fat mass to fat free mass (r_MZ_ = 0.69, 95% CI: 0.42–0.85, *n* = 36) (Fig. 2), where “n” represents the total number of twin pairs from all studies. There were significant pooled r_MZ_ for fat mass (r_MZ_ = 0.58, 95% CI: 0.13–0.83, *n* = 48), fat free mass (r_MZ_ = 0.57, 95% CI: 0.35–0.73, *n* = 73) (Fig. 3), body fat percentage (r_MZ_ = 0.55, 95% CI: 0.32–0.72, *n* = 72), waist circumference (r_MZ_ = 0.50, 95% CI: 0.09–0.77, *n* = 27) and hip circumference (r_MZ_ = 0.51, 95% CI: 0.11–0.77, *n* = 27) (Fig. 4). However, the pooled r_MZ_ was lower and not statistically significantly different from 0 for waist-to-hip ratio (r_MZ_ = 0.29, 95% CI: −0.16–0.64, *n* = 27) (Fig. 5).

**Table 4:** Pooled within monozygotic (MZ) twin pair correlations (95% confidence intervals)

Outcome	All studies	Studies including a combined physical activity and diet intervention	Studies only including a physical activity intervention
Body fat percentage (%)	0.55 (0.32–0.72)*** (*n* = 72) Poehlam A et al. (1987) Koenigstorfer J et al. (2011) Hopkins N et al. (2012) Bouchard C et al. (1994) Hainer V et al. (2000) Hamel P et al. (1986) Prud’Homme D et al. (1984) Afman G et al. (1988)	0.61 (0.28–0.82)** (*n* = 33) Poehlam A et al. (1987) Koenigstorfer J et al. (2011) Bouchard C et al. (1994) Hainer V et al. (2000)	0.49 (0.16–0.73)** (*n* = 39) Hopkins N et al. (2012) Hamel P et al. (1986) Prud’Homme D et al. (1984) Afman G et al. (1988)
BMI	0.69 (0.49–0.82)*** (*n* = 58) Koenigstorfer J et al. (2011) Hopkins N et al. (2012) Bouchard C et al. (1994) Hainer V et al. (2000) Prud’Homme D et al. (1984) Afman G et al. (1988)	0.79 (0.54–0.91)*** (*n* = 27) Koenigstorfer J et al. (2011) Bouchard C et al. (1994) Hainer V et al. (2000)	0.58 (0.23–0.79)** (*n* = 31) Hopkins N et al. (2012) Prud’Homme D et al. (1984) Afman G et al. (1988)
Fat free mass (kg)	0.57 (0.35–0.73)*** (*n* = 73) Poehlam A et al. (1987) Koenigstorfer J et al. (2011) Hopkins N et al. (2012) Bouchard C et al. (1994) Hainer V et al. (2000) Hamel P et al. (1986) Prud’Homme D et al. (1984) Afman G et al. (1988)	0.71 (0.43–0.87)*** (*n* = 33) Poehlam A et al. (1987) Koenigstorfer J et al. (2011) Bouchard C et al. (1994) Hainer V et al. (2000)	0.43 (0.09–0.68)* (*n* = 40) Hopkins N et al. (2012) Hamel P et al. (1986) Prud’Homme D et al. (1984) Afman G et al. (1988)
Fat mass (kg)	0.58 (0.13–0.83)* (*n* = 48) Poehlam A et al. (1987) Koenigstorfer J et al. (2011) Bouchard C et al. (1994) Hainer V et al. (2000) Hamel P et al. (1986) Prud’Homme D et al. (1984)	0.68 (0.16–0.90)* (*n* = 33) Poehlam A et al. (1987) Koenigstorfer J et al. (2011) Bouchard C et al. (1994) Hainer V et al. (2000)	0.27 (−0.36–0.73) (*n* = 15) Hamel P et al. (1986) Prud’Homme D et al. (1984)
Fat mass to fat free mass ratio	0.69 (0.42–0.85)*** (*n* = 36) Bouchard C et al. (1994) Hainer V et al. (2000) Hamel P et al. (1986) Prud’Homme D et al. (1984)	0.82 (0.58–0.93)*** (*n* = 21) Bouchard C et al. (1994) Hainer V et al. (2000)	0.30 (−0.33–0.75) (*n* = 15) Hamel P et al. (1986) Prud’Homme D et al. (1984)
Waist circumference (cm)	0.50 (0.09–0.77)* (*n* = 27) Koenigstorfer J et al. (2011) Bouchard C et al. (1994) Hainer V et al. (2000)		–
Hip circumference (cm)	0.51 (0.11–0.77)* (*n* = 27) Koenigstorfer J et al. (2011) Bouchard C et al. (1994) Hainer V et al. (2000)		–
Waist to hip ratio	0.29 (−0.16–0.64) (*n* = 27) Koenigstorfer J et al. (2011) Bouchard C et al. (1994) Hainer V et al. (2000)		–
Sum of skin folds (cm)	0.67 (0.37–0.85)*** (*n* = 30) Bouchard C et al. (1994) Hainer V et al. (2000) Prud’Homme D et al. (1984)	0.73 (0.39–0.89)*** (*n* = 21) Bouchard C et al. (1994) Hainer V et al. (2000)	0.49 (−0.26–0.87) (*n* = 9) Prud’Homme D et al. (1984)
Trunk fat	0.52 (0.12–0.78)* (*n* = 27) Hopkins N et al. (2012) Bouchard C et al. (1994) Hainer V et al. (2000)	0.56 (0.13–0.82)* (*n* = 21) Bouchard C et al. (1994) Hainer V et al. (2000)	0.30 (−0.68–0.89) (*n* = 6) Hopkins N et al. (2012)
Extremity skin fold (cm)	0.54 (−0.39–0.92) (*n* = 21) Bouchard C et al. (1994) Hainer V et al. (2000)		–
Trunk to extremity ratio	0.48 (−0.30–0.88) (*n* = 21) Bouchard C et al. (1994) Hainer V et al. (2000)		–
Weight (kg)	0.67 (0.48–0.79)*** (*n* = 73) Poehlam A et al. (1987) Koenigstorfer J et al. (2011) Hopkins N et al. (2012) Bouchard C et al. (1994) Hainer V et al. (2000) Hamel P et al. (1986) Prud’Homme D et al. (1984) Afman G et al. (1988)	0.73 (0.47–0.88)*** (*n* = 33) Poehlam A et al. (1987) Koenigstorfer J et al. (2011) Bouchard C et al. (1994) Hainer V et al. (2000)	0.61 (0.32–0.79)*** (*n* = 40) Hopkins N et al. (2012) Hamel P et al. (1986) Prud’Homme D et al. (1984) Afman G et al. (1988)
Absolute VO_2_ max (L.min^−1^)	0.38 (0.04–0.64)* (*n* = 42) Bouchard C et al. (1994) Hamel P et al. (1986) Prud’Homme D et al. (1984) Afman G et al. (1988)	0.52 (−0.38–0.92) (*n* = 7) Bouchard C et al. (1994)	0.36 (−0.01–0.64) (*n* = 35) Hamel P et al. (1986) Prud’Homme D et al. (1984) Afman G et al. (1988)
Relative VO_2_ max (mL.min^−1^.kg^−1^)	0.39 (0.07–0.64)* (*n* = 48) Hopkins N et al. (2012) Bouchard C et al. (1994) Hamel P et al. (1986) Prud’Homme D et al. (1984) Afman G et al. (1988)	0.48 (−0.43–0.91) (*n* = 7) Bouchard C et al. (1994)	0.38 (0.04–0.64)* (*n* = 41) Hopkins N et al. (2012) Hamel P et al. (1986) Prud’Homme D et al. (1984) Afman G et al. (1988)

*n* number of twin pairs, *VO*_*2*_ *max* maximal oxygen uptake, *BMI* body mass index

*p < 0.05; **p < 0.01; ***p < 0.001

**Fig. 6:**
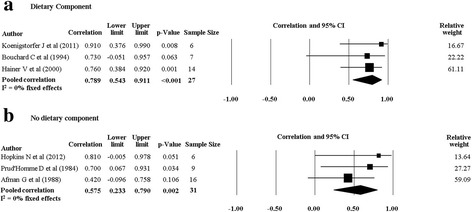
Pooled within monozygotic (MZ) twin pair correlations for BMI in response to physical activity combined with diet, and physical activity without a dietary component. CI: confidence interval; sample size; number of twin pairs

**Fig. 7:**
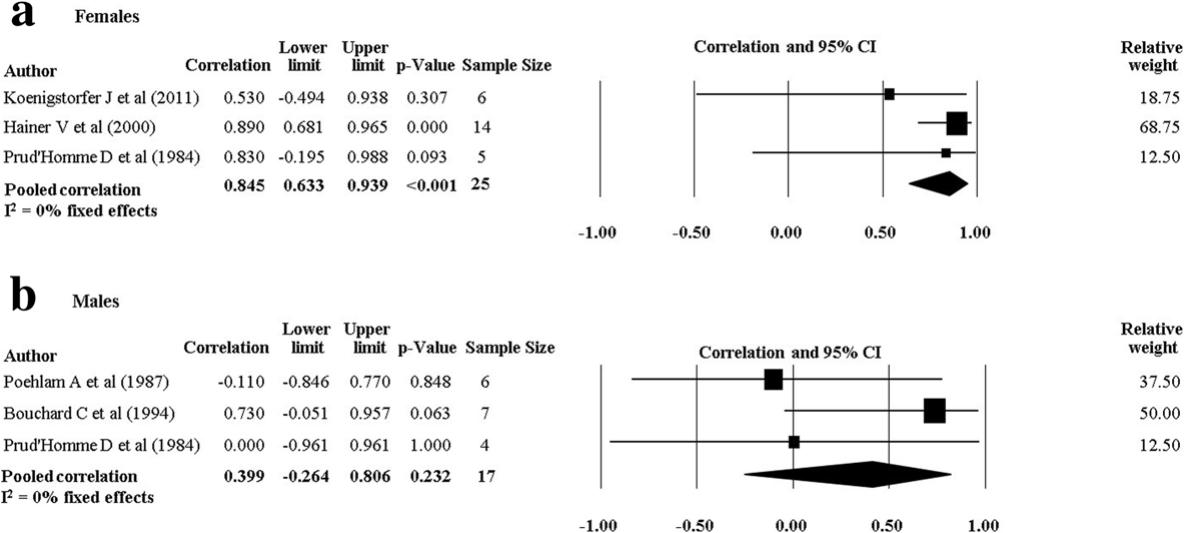
Pooled within monozygotic (MZ) twin pair correlations for fat mass in response to physical activity for females and males. CI: confidence interval; sample size; number of twin pairs

**Fig. 8:**
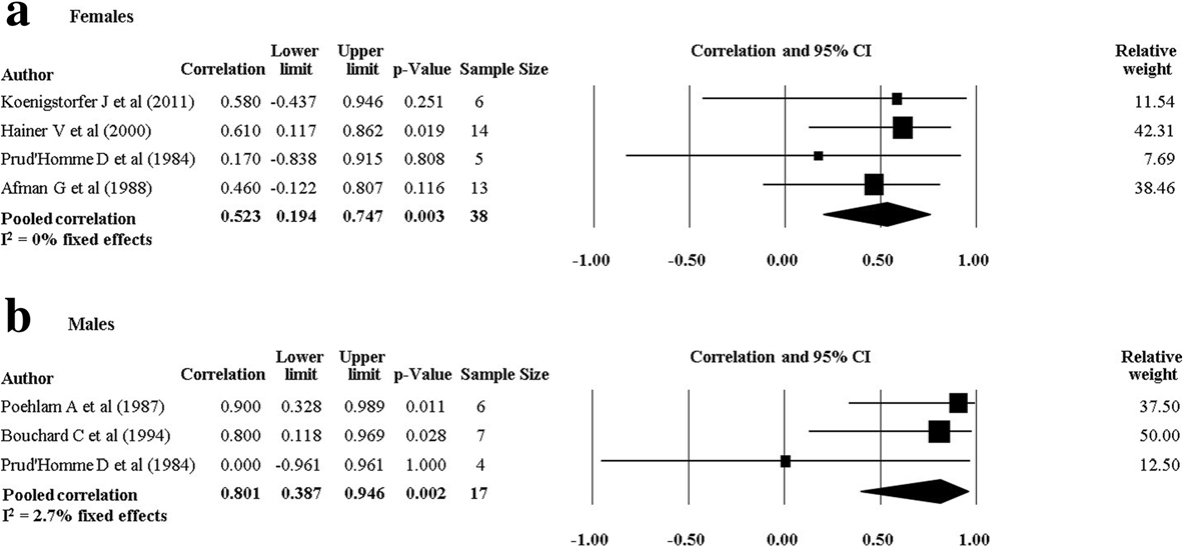
Pooled within monozygotic (MZ) twin pair correlations for fat free mass in response to physical activity for females and males. CI: confidence interval; sample size; number of twin pairs

When we pooled data from twin studies that included a combined PA and diet intervention (four studies [20, 28, 30, 33], there was a trend for the r_MZ_ to be higher across all measures of body composition compared to twin studies that only involved a PA intervention (four studies [29, 32, 34, 35]) (Table 4). The r_MZ_ for BMI was higher when results were pooled for studies including a combined PA and diet intervention (r_MZ_ = 0.79, 95% CI: 0.54–0.91, *n* = 27), compared to studies only involving a PA intervention (r_MZ_ = 0.58, 95% CI: 0.23–0.79, *n* = 31) (Fig. 6), although confidence intervals were wide. Meta-analyses for each outcome were stratified by gender. The r_MZ_ was variable between males and females, depending on the outcome assessed (Table 5), with wide confidence intervals observed for both males and females. The pooled r_MZ_ for the response of fat mass following PA was higher and statistically significant in females (r_MZ_ = 0.85, 95% CI: 0.63–0.94, *n* = 25) compared to males (r_MZ_ = 0.40, 95% CI: −0.26–0.81, *n* = 17) (Fig. 7). However, the pooled r_MZ_ for fat free mass was higher in males (r_MZ_ = 0.80, 95% CI: 0.39–0.95, *n* = 17) compared to females (r_MZ_ = 0.52, 95% CI: 0.19–0.75, *n* = 38) (Fig. 8), both being statistically significantly different from 0 but not from each other.

**Table 5:** Pooled within monozygotic (MZ) twin pair correlations (95% confidence intervals)

Outcome	All studies	Females	Males
Body fat percentage (%)	0.55 (0.32–0.72)*** (*n* = 72) Poehlam A et al. (1987) Koenigstorfer J et al. (2011) Hopkins N et al. (2012) Bouchard C et al. (1994) Hainer V et al. (2000) Hamel P et al. (1986) Prud’Homme D et al. (1984) Afman G et al. (1988)	0.63 (0.36–0.80)*** (*n* = 41) Koenigstorfer J et al. (2011) Hainer V et al. (2000) Prud’Homme D et al. (1984) Afman G et al. (1988)	0.58 (−0.04–0.87) (*n* = 17) Poehlam A et al. (1987) Bouchard C et al. (1994) Prud’Homme D et al. (1984)
BMI	0.69 (0.49–0.82)*** (*n* = 58) Koenigstorfer J et al. (2011) Hopkins N et al. (2012) Bouchard C et al. (1994) Hainer V et al. (2000) Prud’Homme D et al. (1984) Afman G et al. (1988)	0.63 (0.36–0.80)*** (*n* = 41) Koenigstorfer J et al. (2011) Hainer V et al. (2000) Prud’Homme D et al. (1984) Afman G et al. (1988)	0.63 (−0.13–0.93) (*n* = 11) Bouchard C et al. (1994) Prud’Homme D et al. (1984)
Fat free mass (kg)	0.57 (0.35–0.73)*** (*n* = 73) Poehlam A et al. (1987) Koenigstorfer J et al. (2011) Hopkins N et al. (2012) Bouchard C et al. (1994) Hainer V et al. (2000) Hamel P et al. (1986) Prud’Homme D et al. (1984) Afman G et al. (1988)	0.52 (0.19–0.75)** (*n* = 38) Koenigstorfer J et al. (2011) Hainer V et al. (2000) Prud’Homme D et al. (1984) Afman G et al. (1988)	0.80 (0.39–0.95)** (*n* = 17) Poehlam A et al. (1987) Bouchard C et al. (1994) Prud’Homme D et al. (1984)
Fat mass (kg)	0.58 (0.13–0.83)* (*n* = 48) Poehlam A et al. (1987) Koenigstorfer J et al. (2011) Bouchard C et al. (1994) Hainer V et al. (2000) Hamel P et al. (1986) Prud’Homme D et al. (1984)	0.85 (0.63–0.94)*** (*n* = 25) Koenigstorfer J et al. (2011) Hainer V et al. (2000) Prud’Homme D et al. (1984)	0.40 (−0.26–0.81) (*n* = 17) Poehlam A et al. (1987) Bouchard C et al. (1994) Prud’Homme D et al. (1984)
Fat mass to fat free mass ratio	0.69 (0.42–0.85)*** (*n* = 36) Bouchard C et al. (1994) Hainer V et al. (2000) Hamel P et al. (1986) Prud’Homme D et al. (1984)	0.85 (0.61–0.95)*** (*n* = 19) Hainer V et al. (2000) Prud’Homme D et al. (1984)	0.62 (−0.15–0.92) (*n* = 11) Bouchard C et al. (1994) Prud’Homme D et al. (1984)
Waist circumference (cm)	0.50 (0.09–0.77)* (*n* = 27) Koenigstorfer J et al. (2011) Bouchard C et al. (1994) Hainer V et al. (2000)	0.36 (−0.15–0.72) (*n* = 20) Koenigstorfer J et al. (2011) Hainer V et al. (2000)	0.83 (0.21–0.97)* (*n* = 7) Bouchard C et al. (1994)
Hip circumference (cm)	0.51 (0.11–0.77)* (*n* = 27) Koenigstorfer J et al. (2011) Bouchard C et al. (1994) Hainer V et al. (2000)	0.58 (0.13–0.83)* (*n* = 20) Koenigstorfer J et al. (2011) Hainer V et al. (2000)	0.25 (−0.62–0.84) (*n* = 7) Bouchard C et al. (1994)
Waist to hip ratio	0.29 (−0.16–0.64) (*n* = 27) Koenigstorfer J et al. (2011) Bouchard C et al. (1994) Hainer V et al. (2000)	0.27 (−0.24–0.66) (*n* = 20) Koenigstorfer J et al. (2011) Hainer V et al. (2000)	0.35 (−0.55–0.87) (*n* = 7) Bouchard C et al. (1994)
Sum of skin folds (cm)	0.67 (0.37–0.85)*** (*n* = 30) Bouchard C et al. (1994) Hainer V et al. (2000) Prud’Homme D et al. (1984)	0.78 (0.46–0.92)*** (*n* = 19) Hainer V et al. (2000) Prud’Homme D et al. (1984)	0.51 (−0.30–0.89) (*n* = 11) Bouchard C et al. (1994) Prud’Homme D et al. (1984)
Trunk fat	0.52 (0.12–0.78)* (*n* = 27) Hopkins N et al. (2012) Bouchard C et al. (1994) Hainer V et al. (2000)	0.67 (0.22–0.89)** (*n* = 14) Hainer V et al. (2000)	0.15 (−0.68–0.81) (*n* = 7) Bouchard C et al. (1994)
Extremity skin fold (cm)	0.54 (−0.39–0.92) (*n* = 21) Bouchard C et al. (1994) Hainer V et al. (2000)	0.78 (0.43–0.93)** (*n* = 14) Hainer V et al. (2000)	0.00 (−0.75–0.75) (*n* = 7) Bouchard C et al. (1994)
Trunk to extremity ratio	0.48 (−0.30–0.88) (*n* = 21) Bouchard C et al. (1994) Hainer V et al. (2000)	0.70 (0.27–0.90)** (*n* = 14) Hainer V et al. (2000)	0.00 (−0.75–0.75) (*n* = 7) Bouchard C et al. (1994)
Weight (kg)	0.67 (0.48–0.79)*** (*n* = 73) Poehlam A et al. (1987) Koenigstorfer J et al. (2011) Hopkins N et al. (2012) Bouchard C et al. (1994) Hainer V et al. (2000) Hamel P et al. (1986) Prud’Homme D et al. (1984) Afman G et al. (1988)	0.70 (0.46–0.84)*** (*n* = 41) Koenigstorfer J et al. (2011) Hainer V et al. (2000) Prud’Homme D et al. (1984) Afman G et al. (1988)	0.45 (−0.20–0.83) (*n* = 17) Poehlam A et al. (1987) Bouchard C et al. (1994) Prud’Homme D et al. (1984)
Absolute VO_2_ max (L.min^−1^)	0.38 (0.04–0.64)* (*n* = 42) Bouchard C et al. (1994) Hamel P et al. (1986) Prud’Homme D et al. (1984) Afman G et al. (1988)	0.74 (−0.18–0.97) (*n* = 21) Prud’Homme D et al. (1984) Afman G et al. (1988)	0.49 (−0.33–0.89) (*n* = 11) Bouchard C et al. (1994) Prud’Homme D et al. (1984)
Relative VO_2_ max (mL.min^−1^.kg^−1^)	0.39 (0.07–0.64)* (*n* = 48) Hopkins N et al. (2012) Bouchard C et al. (1994) Hamel P et al. (1986) Prud’Homme D et al. (1984) Afman G et al. (1988)	0.51 (0.06–0.79)* (*n* = 21) Prud’Homme D et al. (1984) Afman G et al. (1988)	0.40 (−0.43–0.86) (*n* = 11) Bouchard C et al. (1994) Prud’Homme D et al. (1984)

*n* number of twin pairs, *VO*_*2*_ *max* maximal oxygen uptake, *BMI* body mass index

*p < 0.05; **p < 0.01; ***p < 0.001

We were able to extract heritability, and maximal heritability estimates for measures of body composition from three twin studies [27, 29, 32] (with raw data used to generate heritability estimates from one [29]), and two family studies, respectively [24, 36]. However, we did not report the heritability estimates from two twin studies [29, 32], as there were no statistically significant differences between the r_MZ_ and r_DZ_, making the estimates uninformative (Table 1). Danis and colleagues [27] used different methodology to calculate heritability and we reported the estimates in Table 1. Maximal heritability estimates ranged from 0–21% in family studies, with higher estimates for trunk and extremity skin folds compared to measures of fat mass and waist circumference (Table 6).

**Table 6:** Maximal heritability estimates from family studies (includes variance explained by genetic and non-genetic sources shared within families)

Outcome	Author (year)	Maximal heritability (95% CI)
Fat mass (kg)	Rice T (1999)	0%^a^
Trunk skin folds (cm)	Perusse L (2000)	21% (14 to 28%)
Extremity skin folds (cm)	Perusse L (2000)	15% (5 to 25%)
Subcutaneous fat (sum of eight skin folds) (cm)	Perusse L (2000)	15% (8 to 22%)
Trunk to extremity skin fold ratio (adjusted for subcutaneous fat)	Perusse L (2000)	14% (10 to 18%)
Waist circumference (cm) (adjusted for BMI)	Perusse L (2000)	0%^a^
Absolute VO_2_ max (L.min^−1^)	Bouchard C (1999)	47%^a^
Absolute VO_2_ max at ventilatory threshold (L.min^−1^)	Gaskill SE (2001)	Caucasian: 22% (−2 to 46%) African-American: 51 (27% to 75%)
Relative VO_2_ max (mL.min^−1^.kg^−1^)	Perusse L (2001)	50 W: 57%^a^
		60% VO_2_ max: 23%^a^
		80% VO_2_ max: 44%^a^

*CI* confidence interval, *VO*_*2*_ *max* maximal oxygen uptake, *W* watts, *BMI* body mass index

^a^Unable to calculate the standard error and thus present the 95% CI

### Outcomes of Cardiorespiratory Fitness

There were nine studies (six twin studies [27, 29, 30, 32, 34, 35] and three family studies [19, 37, 38]) which investigated cardiorespiratory fitness measures and their response following a PA intervention. Pooling of five twin studies results (excluding Danis and colleagues [27] due to different methodology) suggests there are significant pooled r_MZ_ for absolute VO_2_max (L.min^−1^) (r_MZ_ = 0.38, 95% CI: 0.04–0.64, *n* = 42) and relative VO_2_max (mL.min^−1^.kg^−1^) (r_MZ_ = 0.39, 95% CI: 0.07–0.64, *n* = 48) (Table 4).

**Fig. 9:**
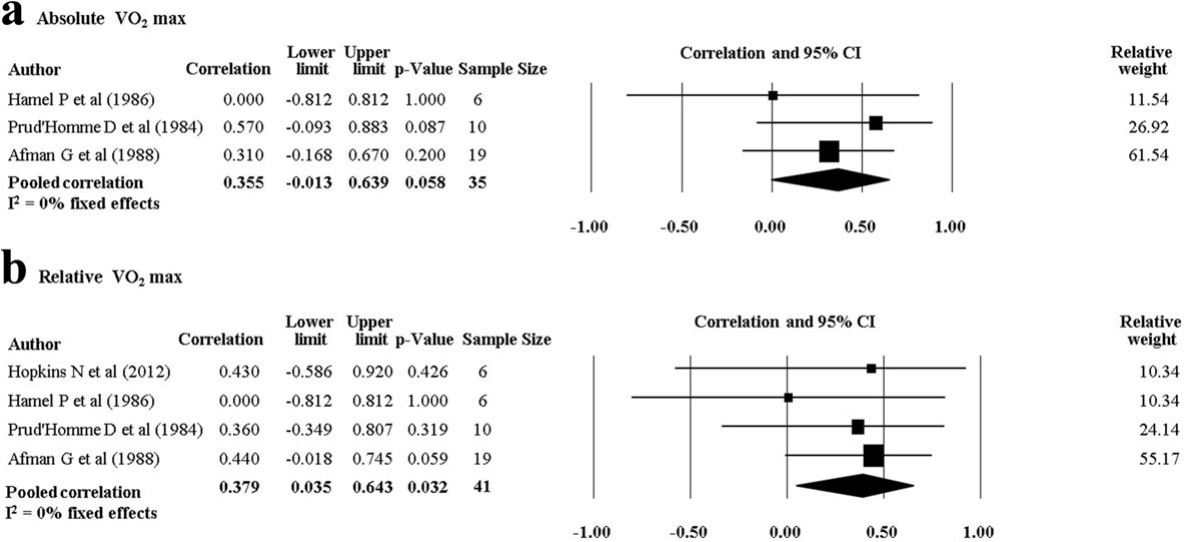
Pooled within monozygotic (MZ) twin pair correlations for absolute and relative maximal oxygen uptake (VO_2_ max) in response to physical activity without a dietary component. CI: confidence interval; sample size; number of twin pairs

**Fig. 10:**
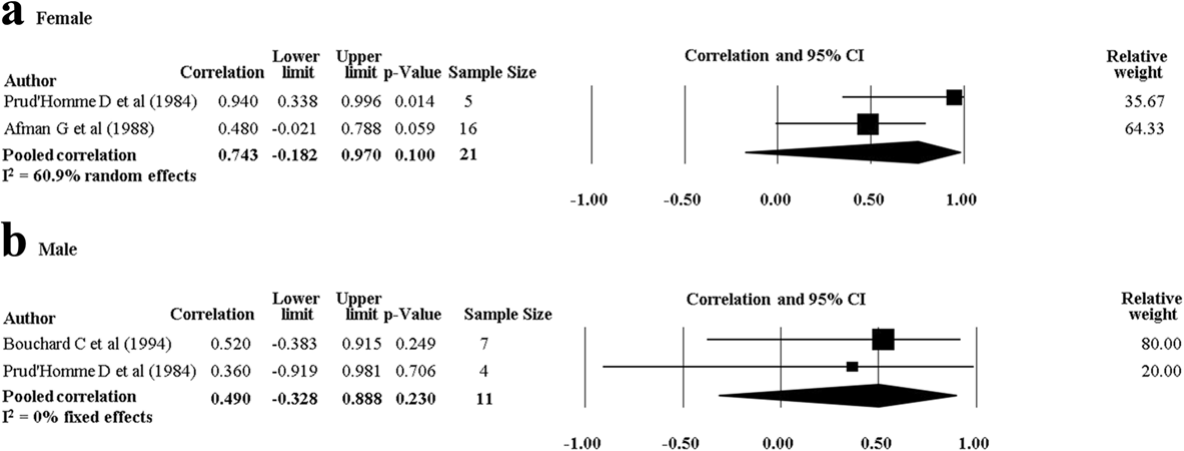
Pooled within monozygotic (MZ) twin pair correlations for absolute maximal oxygen uptake (VO_2_ max) in response to physical activity for females and males. CI: confidence interval; sample size; number of twin pairs

There was one twin study which investigated the response of cardiorespiratory fitness following a combined PA and diet intervention [30] and four twin studies which investigated the response of cardiorespiratory fitness following an isolated PA intervention [29, 32, 34, 35]. The r_MZ_ for absolute and relative VO_2_max in the study (*n* = 7) which combined PA with diet (r_MZ_ = 0.52, 95% CI: −0.38–0.92, and r_MZ_ = 0.48, 95% CI: −0.43–0.91, respectively) was higher than the pooled r_MZ_ from the studies which only investigated a PA intervention (r_MZ_ = 0.36, 95% CI: −0.01–0.64, *n* = 35, and r_MZ_ = 0.38, 95% CI: 0.04–0.64, *n* = 41, respectively) (Fig. 9) although the confidence intervals overlapped, and the r_MZ_ from the individual study was not statistically significantly different from 0 (with 95% CIs generated from the meta-analysis software). Meta-analyses for absolute and relative VO_2_max were stratified by gender, with the pooled r_MZ_ being higher in females (Table 5). The pooled r_MZ_ for the response of absolute VO_2_max following PA was 0.74 in females (*n* = 21) and 0.49 in males (*n* = 11), although neither were statistically significantly different from 0 (Fig. 10).

Heritability estimates for the response of VO_2_max from two twin studies [29, 32] were not reported, as there were no statistically significant differences between the r_MZ_ and r_DZ_ (Table 1). Danis and colleagues [27] used different methodology to calculate heritability and we reported the estimates in Table 1 .Maximal heritability estimates from the three included family studies [19, 37, 38] were variable, ranging from 22–57% depending on race and when VO_2_max was measured (e.g., ventilatory threshold, pre-determined power levels, etc.) (Table 6).

## Discussion

Our results demonstrate consistent evidence that shared familial factors (whether genetic or environmental) play a role in the response of body composition and cardiorespiratory fitness following PA, despite varying on the outcome being assessed, particularly when results were stratified by gender. The pooled r_MZ_ were generally >0.5, and the bulk of most CIs also exceeded 0.5. Shared familial factors appear to play a larger role in the response of body composition when compared to cardiorespiratory fitness, and may have more influence on the response for most outcomes when considering a combined PA and diet intervention.

### Heritability Estimates and the Within MZ Twin Pair Correlation

Only a few studies included DZ twins (*n* = 2) [29, 32], so we pooled the r_MZ_ to provide an estimate of the upper bound of heritability. Traditionally, twin and family studies investigating the heritability of a phenotype (e.g., PA engagement [15, 16], BMI [39], and chronic pain [40]) have done so using a cross-sectional design, with twin studies dividing the variance of a phenotype into components or proportions due to additive genetic factors (heritability), shared environmental factors, and unique environmental factors. Our pooled estimates represent the upper bound of heritability, including variance from additive genetic and shared environmental factors. However, our study investigated how shared familial factors influence the response to PA, with the r_MZ_ derived from the change in outcome status following an intervention. Since interventions were implemented over a specified timeframe, with training parameters controlled, it has been suggested that unique and shared environmental factors would make minor contributions to the variance of the response to PA [22], resulting in a r_MZ_ that would give a close estimate of heritability. However, family studies included in this review found significant correlations between spouses for the response of body composition [38] and cardiorespiratory fitness [19, 24] following a PA intervention. Although some suggest this indicates a greater influence of shared environmental factors [19], this correlation may equally be due to shared genes (assortative mating), so without making strong assumptions as to which is occurring in spouses, this is unlikely to indicate a greater influence of shared environmental factors.

### Shared Familial Influence on Changes of Body Composition

Factors shared within MZ twin pairs appear to play a strong role in the response of BMI (pooled r_MZ_ = 0.69) following a PA intervention (Fig. 2), although they appear to be less influential in the response of other outcomes (e.g., waist-to-hip ratio) (Fig. 5). Although we were unable to pool heritability estimates, our pooled r_MZ_ for the response of BMI following PA appears to be within the range of previous studies reporting the cross-sectional heritability of BMI (ranging from 47–90% in twins studies [39]). However, other cross-sectional studies have reported heritability estimates for waist circumference (66%) and body fat percentage (68%) [41] that appear to be slightly higher than our r_MZ_ in response to exercise (pooled r_MZ_ = 0.50 and 0.55, respectively), especially considering our results represent the upper bound of heritability. Therefore, by comparing our results to those of previous investigations, it appears the genetic influences on an individual’s body composition (cross-sectional association) might be different, and perhaps higher, than the way their body composition responds to PA.

Previous cross-sectional twin studies have reported gender differences for the heritability of body composition, although they appear to vary depending on the outcome of interest. A twin study by Schousboe and colleagues [42] reported that males have higher heritability estimates compared to females for body fat percentage (63 and 59%, respectively), sum of skin folds (65 and 61%, respectively), waist circumference (61 and 48%, respectively) and waist-to-hip ratio (22 and 10%, respectively). However, other studies have reported higher heritability estimates in females across a variety of body composition measures [43, 44]. The variability between genders for the heritability of body composition has been supported in various twin studies, regardless of the sample size, methods of analyses or ethnicity [43, 45–47]. Our results extend the understanding that gender influences the role shared familial factors, including genes, play in the variation of body composition (cross-sectional association), and suggests gender influences how shared familial factors influence the response of body composition measures following PA. In particular, shared familial factors appear to have a greater influence on changes in fat mass for females engaged in PA (Fig. 7) and fat free mass for males engaged in PA (Fig. 8). Therefore, to better understand how both genetics and shared environmental factors impact an individual’s response to PA, it may be important to take into consideration the gender of the individual, and the outcome of interest.

### Shared Familial Influence on Changes in Cardiorespiratory Fitness

The heritability of VO_2_max assessed in cross-sectional studies ranges from 40–71% in twin studies [41, 48] and has been reported at 50% (maximal heritability) in the HERITAGE Family Study [49]. Our pooled r_MZ_ were 0.38 and 0.39 for absolute and relative VO_2_max, respectively, and appear to be smaller than heritability estimates for an individual’s pre-training VO_2_max, although the CIs for our results include the cross-sectional estimates. This suggests genetics may be more influential in determining an individual’s cardiorespiratory fitness, compared to their fitness response following PA, although the biological explanation for this is unclear.

The point estimates of the r_MZ_ for the response of cardiorespiratory fitness following PA appear slightly greater in females (Fig. 10), although the CIs for both the male and female correlations cover almost all the possible range of values due to small sample sizes in the original studies. Similarly, existing studies investigating the heritability of cardiorespiratory fitness have been limited in their ability to analyze the effect of gender due to small sample sizes [41], and single gender cohorts [48]. Therefore, our results should be viewed as preliminary with this area deserving attention in future studies.

### Strengths and Limitations

Our study demonstrated considerable strengths in its design. First, previous studies have predominantly focussed on investigating the heritability of PA engagement (cross-sectional association) [15], without considering how genetics and shared environmental factors impact an individual’s response to PA. From a health-care perspective, it may be more important to investigate how genetics and environmental factors influence the response to PA. It is likely the response to PA would be more dependent on unique environmental factors, such as training parameters (frequency, intensity, duration, type), adherence, therapeutic alliance, and many more. However, neither training frequency nor duration appeared to influence the r_MZ_ for either body composition or cardiorespiratory fitness, which may suggest the role genetics plays in response to PA is independent of these parameters. Quantifying the influence of genetics and environmental factors on the response to PA may serve to explain why certain individuals do not respond as well to a structured PA program across a variety of outcomes, with implications for how we can modify the training environment to achieve a positive response. Second, twin studies which have investigated how genetics influence the response to PA have been limited in their ability to draw firm conclusions due to small sample sizes. Small sample sizes of the included studies explain cases where our pooled CIs were wide, even though we were able to pool results for up to 83 MZ twin pairs, improving the precision around these estimates. To obtain 95% CIs of sufficiently small width to be informative (e.g., a total width of 0.1), in studies that include only MZ twins, approximately 400 twin pairs are required if the correlation is moderately high (0.7), and greater than 1000 twin pairs if the correlation is 0.4. For studies including both MZ and DZ twins, 150 twin pairs of each zygosity would be required to detect a significant difference (*p* = 0.05) between r_MZ_ = 0.7 and r_DZ_ = 0.5, with 80% power. If both correlations are lower (e.g., r_MZ_ = 0.5 and r_DZ_ = 0.3), 275 twins pairs of each zygosity would be required. Many of the studies which reported heritability or maximal heritability also failed to report confidence intervals for their estimates, or provide sufficient information to enable these to be estimated accurately (Tables 1 and 6). Although point estimates are available, there is clearly a substantial information difference between a heritability of 47% with a 95% CI of 44–50% and the same heritability with a 95% CI of 10–85%, and we expect that studies included in this review are more like to the second situation, limiting the utility of the reported estimates. Third, raw data were used to re-analyse previously reported correlations in four twin studies [29, 30, 34, 35] and adjust for age, gender (if applicable), and baseline values. This provided a more precise estimate for quantifying the role genetics plays in the response to PA.

Our study has a few limitations which need to be considered when interpreting the results. First, samples from included twin studies differed in their age, baseline values, PA interventions, and diet interventions. Furthermore, one study recruited twin pairs admitted to an obesity unit for a 40-day physical activity and diet program [28], a sample not representative of the general population. However, we conducted a number of sensitivity analyses and the exclusion of any single study did not significantly affect the results for any of the outcomes (Additional file 2). In addition, we performed separate meta-analyses for studies which included a diet intervention, to better understand how the difference between interventions impacted our results. Second, two twin studies [29, 30] reported drop outs on the basis of twin pairs failing to complete the training protocol (Table 2), while the family studies only analyzed data from participants who completed the training protocol (Table 3). We acknowledge that this may limit the generalizability of the results, as participants who completed the training protocol are likely to be more motivated to engage in PA than the general population. Third, although using a classical twin design to estimate heritability is a widely reported method to investigate how genetics contributes to the variation of a phenotype, it does have some limitations, and together with the fact that individual twin studies had small sample sizes, is the reason we did not focus our results on these estimates. The use of self-reported zygosity measures, based on the difficulty of being told apart by parents, is often criticized. MZ twins who differ in their height and weight can be mistakenly classified as DZ twins when using self-reported measures, resulting in an underestimation of heritability [50]. However, only one study included in this review assessed zygosity using only a self-reported questionnaire [32], with another failing to describe how zygosity was assessed [20]. The remaining twin studies (*n* = 7) verified questionnaire-based zygosity through DNA mapping. In addition, not considering the genotype-environment interaction is a limitation of the classical twin design, since genetic factors can influence an individual’s choice/exposure to the environment. However, studies included in this review utilized a controlled training environment, reducing the likelihood that an individual’s genetics would impact their environment for the experimental period. Furthermore, the use of heritability as a measure, although widely reported, has some limitations; it is dependent on the modeling of the mean, on the amount of variance and measurement error (which may be larger in studies of changes in outcomes compared with cross-sectional studies of outcomes [51]) and on the total variation within a population, which may differ between populations and between the same population measured at different times [52]. Finally, when estimated from classic twin studies, this estimate depends on the assumption that environments are shared to the same extent by MZ and DZ pairs—an assumption that is rarely considered or tested in practice [53].

### Clinical Implications

The results of this current investigation are consistent with a substantial influence of genes on the response of body composition and cardiorespiratory fitness following PA. These results have implications for conditions which utilize PA as a management strategy, for example, diabetes, and low back pain. If an individual’s response to a PA intervention is partially dictated by genetic factors this could potentially explain why some individuals fail to respond to increased PA. This has implications for changing the modifiable training environment to achieve a desired effect (e.g., increased intensity, frequency, or duration), or excluding people who demonstrate a poor response to reduce treatment costs and consumer disappointment. Furthermore, if genetic factors are involved in the poor response to PA as an intervention, this has implications for the selection of alternative management strategies, or a modification to the outcome investigated, since individuals who show a low training response to one parameter (e.g., VO_2_max) might in fact respond positively to another (e.g., BMI).

Research linking genetic markers to a specific phenotype (quantitative trait locus analysis) have aided the mechanistic understanding of how genetics influence the response of body composition and cardiorespiratory fitness following PA, although more genetic research needs to be done. A family study investigated over 300,000 single-nucleotide polymorphisms (SNPs) and identified 21 SNPs which accounted for 49% of the variance in the response of VO_2_max following a PA intervention, with one SNP (rs6552828) accounting for ~6% of the variance [54]. The variance explained by these 21 SNPs is similar to the maximal heritability of VO_2_max response from the family study included in this review (47%), although this study observed significant spouse correlations which some consider consistent with shared environmental effects, thereby reducing the variance explained by genetics [19]. Similarly, nine SNPs were found to explain 20% of the variance of submaximal heart rate in response to PA, with one SNP (rs2253206) accounting for ~5% of the variance [55]. Earlier studies have identified candidate genes that are strongly linked to or associated with the response of BMI, fat mass, fat-free mass, and body fat percentage following a PA intervention [56]. For example, the insulin-like growth factor-1 (IGF-1) gene marker was strongly linked to response of fat-free mass following PA [57], with linkage also present for a polymorphism in the S100A gene [56] (predominantly found in slow-twitch skeletal and cardiac muscle fibers [58]). Research identifying genetic markers is promising and may aid the prediction of how an individual’s body composition and cardiorespiratory fitness will respond following PA, although it is essential these results are replicated in larger samples, and through a variety of genetic analyses before definite conclusions are reached [59, 60]. Furthermore, research investigating practical and cost-effective methods to identify those who will respond positively to a PA intervention would be of significant interest from a public health and clinical perspective. For example, information regarding how family members have previously responded to PA may help to predict how an individual will respond to a similar intervention, potentially reducing the need for costly genetic testing.

## Conclusions

Shared familial factors, including genetics, are likely to be significant contributors to the response of several markers of body composition and cardiorespiratory fitness following PA. Shared familial factors may play a stronger role in the response of body composition when compared to cardiorespiratory fitness, and may be more influential in dictating the response for measures of BMI, fat mass, and body fat percentage, compared to waist-to-hip ratio. The influence shared familial factors have on the response to PA may be different in males and females, with such factors having a greater influence on changes in fat mass for females, and fat-free mass for males. In addition, shared familial factors appear to be more influential in dictating the response of body composition and cardiorespiratory fitness when PA is combined with diet.

These results have implications for the management of conditions which advocate increased levels of PA, since genetic factors might serve as an explanation for why some people respond more effectively than others in specific measures of PA. To further quantify the role genetics and environmental factors play in the response to PA future research should focus on adequately powered studies including both MZ and DZ twins, and the replication of existing genome-wide association studies to identify important genetic markers for the response to PA.

## Additional files

Additional file 1: Search strategy. (DOCX 18 kb)
Additional file 2: Sensitivity analysis excluding one study at a time. (JPG 363 kb)
