# Supplementary material for: The Beneficial Effects of Physical Activity: Is It Down to Your Genes? A Systematic Review and Meta-Analysis of Twin and Family Studies
Source: Sports Med Open. 2017 Jan 10;3:4. doi: 10.1186/s40798-016-0073-9 (PMC5225201; doi:10.1186/s40798-016-0073-9)
Supplement: Additional file 1: — Search strategy. (DOCX 18 kb) [file 40798_2016_73_MOESM1_ESM.docx]

**APPENDIX 1: Search Strategy**

**MEDLINE**

|  | **Searches** |
| --- | --- |
| Physical Activity | 1. “physical activit*”.mp 2. exp Motor Activity/ 3. “plyometric exercise”.mp 4. exp Exercise Therapy/ 5. exp Physical Endurance/ 6. exp Exercise/ 7. exp “Physical Education and Training”/ 8. “physical fitness”.mp 9. “endurance training”.mp 10. “aerobic exercise”.mp 11. exp Physical Exertion/ 12. “resistance training”.mp 13. 1 or 2 or 3 or 4 or 5 or 6 or 7 or 8 or 9 or 10 or 11 or 12 |
| Twin and Family studies | 1. “twin*”.mp 2. exp Twins, Monozygotic/ 3. exp Twins, Dizygotic/ 4. exp Diseases in Twins/ 5. exp Genetics/ 6. exp Genetic Linkage/ 7. “twin stud*”.mp 8. “herita*”.mp 9. “identical twin*”.mp 10. “family resemblance”.mp 11. exp Family Characteristics/ 12. exp Family Relations/ 13. exp Phenotype/ 14. exp Genotype/ 15. 14 or 15 or 16 or 17 or 18 or 19 or 20 or 21 or 22 or 23 or 24 or 25 or 26 or 27 |
|  | 1. 13 and 28 2. Limit 29 to humans |

**CINHAL**

|  | **Searches** |
| --- | --- |
| Physical Activity | 1. MH "Physical Endurance+" 2. “physical activit*” 3. MH "Physical Education and Training+" 4. “physical fitness” 5. MH "Education, Physical Education" 6. MH "Exercise+" 7. “exercise” 8. “motor activity” 9. MH "Therapeutic Exercise+" 10. “endurance training” 11. "resistance training" 12. MH "Aerobic Exercises+" 13. 1 or 2 or 3 or 4 or 5 or 6 or 7 or 8 or 9 or 10 or 11 or 12 |
| Twin and Family studies | 1. "Twin*" 2. “twin stud*” 3. MH "Multiple Offspring+" 4. "monozygotic twin*" 5. “dizygotic twin*” 6. MH "Genetics+" 7. “herita*” 8. MH "Genetic Diseases, X-Linked+" 9. MH "Hereditary Diseases+" 10. MH "Family Characteristics+" 11. MH "Family Relations+" 12. "family resemblance" 13. ”phenotype” 14. ”genotype” 15. 14 or 15 or 16 or 17 or 18 or 19 or 20 or 21 or 22 or 23 or 24 or 25 or 26 or 27 |
|  | 1. 13 and 28 |

**EMBASE**

|  | **Searches** |
| --- | --- |
| Physical Activity | 1. 'physical activity' 2. 'physical exertion' 3. 'motor activity' 4. 'physical fitness' 5. ‘aerobic exercise’ 6. Exercise:de,ab,ti 7. 'endurance training' 8. 'exercise therapy' 9. 'physical education and training' 10. 'resistance training' 11. 1 or 2 or 3 or 4 or 5 or 6 or 7 or 8 or 9 or 10 |
| Twin and Family studies | 1. Heritage:de,ab,ti 2. 'monozygotic twins' 3. 'dizygotic twins' 4. ‘identical twins’ 5. 'genetic linkage' 6. 'family resemblance' 7. 'family relation' 8. 'family characteristics' 9. herita* 10. 'twin study' 11. Twin* 12. 'genetic variability' 13. 'genetic variation' 14. Genetics:de,ab,ti 15. 12 or 13 or 14 or 15 or 16 or 17 or 18 or 19 or 20 or 21 or 22 or 23 or 24 or 25 |
|  | 1. 11 and 26 2. Limit 27 to humans |

**Sports Discuss**

|  | **Searches** |
| --- | --- |
| Physical Activity | 1. "physical activi*" 2. “exercise" 3. “exercise therapy" 4. "physical fitness" 5. "endurance training" 6. "physical exertion" 7. "motor activity" 8. "physical endurance" 9. "physical education and training" 10. “resistance training” 11. “plyometric exercise” 12. “aerobic exercise” 13. 1 or 2 or 3 or 4 or 5 or 6 or 7 or 8 or 9 or 10 or 11 or 12 |
| Twin and Family studies | 1. "twin*" 2. "monozygotic twin*" 3. "dizygotic twin*" 4. "diseases in twins" 5. "genetic*" 6. "genetic linkage" 7. "twin stud*" 8. "herita*" 9. "family characteristics" 10. "family resemblance" 11. "family relations" 12. “identical twin*” 13. “genotype” 14. “phenotype” 15. 14 or 15 or16 or 17 or 18 or 19 or 20 or 21 or 22 or 23 or 24 or 25 or 26 or 27 |
|  | 1. 13 and 28 |

**AMED**

|  | **Searches** |
| --- | --- |
| Physical Activity | 1. “physical activi*”.mp 2. “motor activity”.mp 3. exp Exercise/ 4. “aerobic exercise”.mp 5. “exercise therapy”.mp 6. exp Physical Endurance/ 7. “physical fitness”.mp 8. “endurance training”.mp 9. “physical exertion”.mp 10. “resistance training”.mp 11. 1 or 2 or 3 or 4 or 5 or 6 or 7 or 8 or 9 or 10 |
| Twin and Family studies | 1. “twin*”.mp 2. “monozygotic twin*”.mp 3. “dizygotic twin*”.mp 4. “genetic*”.mp 5. “twin stud*”.mp 6. “herita*”.mp 7. “identical twin*”.mp 8. exp Family Characteristics/ 9. exp Family Relations/ 10. “genotype”.mp 11. “phenotype”.mp 12. 13 or 14 or 15 or 16 or 17 or 18 or 19 or 20 or 21 or 22 |
|  | 1. 11 and 23 |

**PsycINFO**

|  | **Searches** |
| --- | --- |
| Physical Activity | 1. “physical activi*”.mp 2. “motor activity”.mp 3. exp Exercise/ 4. “aerobic exercise”.mp 5. “exercise therapy”.mp 6. “physical endurance”.mp 7. “physical fitness”.mp 8. “endurance training”.mp 9. “physical exertion”.mp 10. “resistance training”.mp 11. 1 or 2 or 3 or 4 or 5 or 6 or 7 or 8 or 9 or 10 |
| Twin and Family studies | 1. “twin*”.mp 2. “monozygotic twin*”.mp 3. “dizygotic twin*”.mp 4. exp Heterozygotic Twins/ 5. exp Genetics/ 6. exp Genetic Linkage/ 7. “twin stud*”.mp 8. “herita*”.mp 9. “identical twin*”.mp 10. “family characteristics”.mp 11. exp Family Relations/ 12. “genotype”.mp 13. “phenotype”.mp 14. 13 or 14 or 15 or 16 or 17 or 18 or 19 or 20 or 21 or 22 or 23 or 24 |
|  | 1. 11 and 25 2. Limit 26 to humans |

**Scopus**

|  | **Searches** |
| --- | --- |
| Physical Activity | 1. TITLE-ABS-KEY("physical activi*") 2. TITLE-ABS-KEY("exercise") 3. TITLE-ABS-KEY("exercise therapy") 4. TITLE-ABS-KEY("physical fitness") 5. TITLE-ABS-KEY("endurance training") 6. TITLE-ABS-KEY("physical exertion") 7. TITLE-ABS-KEY("motor activity") 8. TITLE-ABS-KEY("physical endurance") 9. TITLE-ABS-KEY ("resistance training") 10. TITLE-ABS-KEY ("aerobic exercise") 11. 1 or 2 or 3 or 4 or 5 or 6 or 7 or 8 or 9 or 10 |
| Twin and Family studies | 1. TITLE-ABS-KEY("twin*") 2. TITLE-ABS-KEY("monozygotic twin*") 3. TITLE-ABS-KEY("dizygotic twin*") 4. TITLE-ABS-KEY("genetics") 5. TITLE-ABS-KEY("genetic linkage") 6. TITLE-ABS-KEY("twin stud*") 7. TITLE-ABS-KEY("herita*") 8. TITLE-ABS-KEY("family characteristics") 9. TITLE-ABS-KEY("family resemblance") 10. TITLE-ABS-KEY("family relations") 11. 13 or 14 or 15 or 16 or 17 or 18 or 19 or 20 or 21 |
| Study type | 1. TITLE-ABS-KEY("cohort study") 2. TITLE-ABS-KEY("longitudinal study") 3. TITLE-ABS-KEY(longitudinal) 4. TITLE-ABS-KEY("follow up study") 5. TITLE-ABS-KEY("follow-up study") 6. TITLE-ABS-KEY("prospective study") 7. TITLE-ABS-KEY (“cross-sectional stud*”) 8. TITLE-ABS-KEY (“cross sectional stud*”) 9. 23 or 24 or 25 or 26 or 27 or 28 or 29 or 30 |
|  | 1. 11 and 22 2. 32 and not 31 3. Exclude: “animals” and “animal” |

**Web of Science**

|  | **Searches** |
| --- | --- |
| Physical Activity | \|  \| 1. TS=("physical activi*") 2. TS=("exercise") 3. TS=("exercise therapy") 4. TS=("physical fitness") 5. TS=("endurance training") 6. TS=("physical exertion") 7. TS=("motor activity") 8. TS=("physical endurance") 9. TS=("physical education and training") 10. TS=(“resistance training”) 11. TS=(“aerobic exercise”) 12. 1 or 2 or 3 or 4 or 5 or 6 or 7 or 8 or 9 or 10 or 11 \| \| --- \| --- \| |
| Twins and Family studies | 1. TS=("twin*") 2. TS=("monozygotic twin*") 3. TS=("dizygotic twin*") 4. TS=("diseases in twins") 5. TS=("genetics") 6. TS=("genetic linkage") 7. TS=("twin stud*") 8. TS=("herita*") 9. TS=("family characteristics") 10. TS=("family resemblance") 11. TS=("family relations") 12. 13 or 14 or 15 or 16 or 17 or 18 or 19 or 20 or 21 or 22 or 23 |
| Study Type | 1. 12 and 24 2. TS=(animals) NOT TS=(humans) 3. 25 not 26 |
