# Supplementary figures and images for: The Beneficial Effects of Physical Activity: Is It Down to Your Genes? A Systematic Review and Meta-Analysis of Twin and Family Studies
Source: Sports Med Open. 2017 Jan 10;3:4. doi: 10.1186/s40798-016-0073-9 (PMC5225201; doi:10.1186/s40798-016-0073-9)

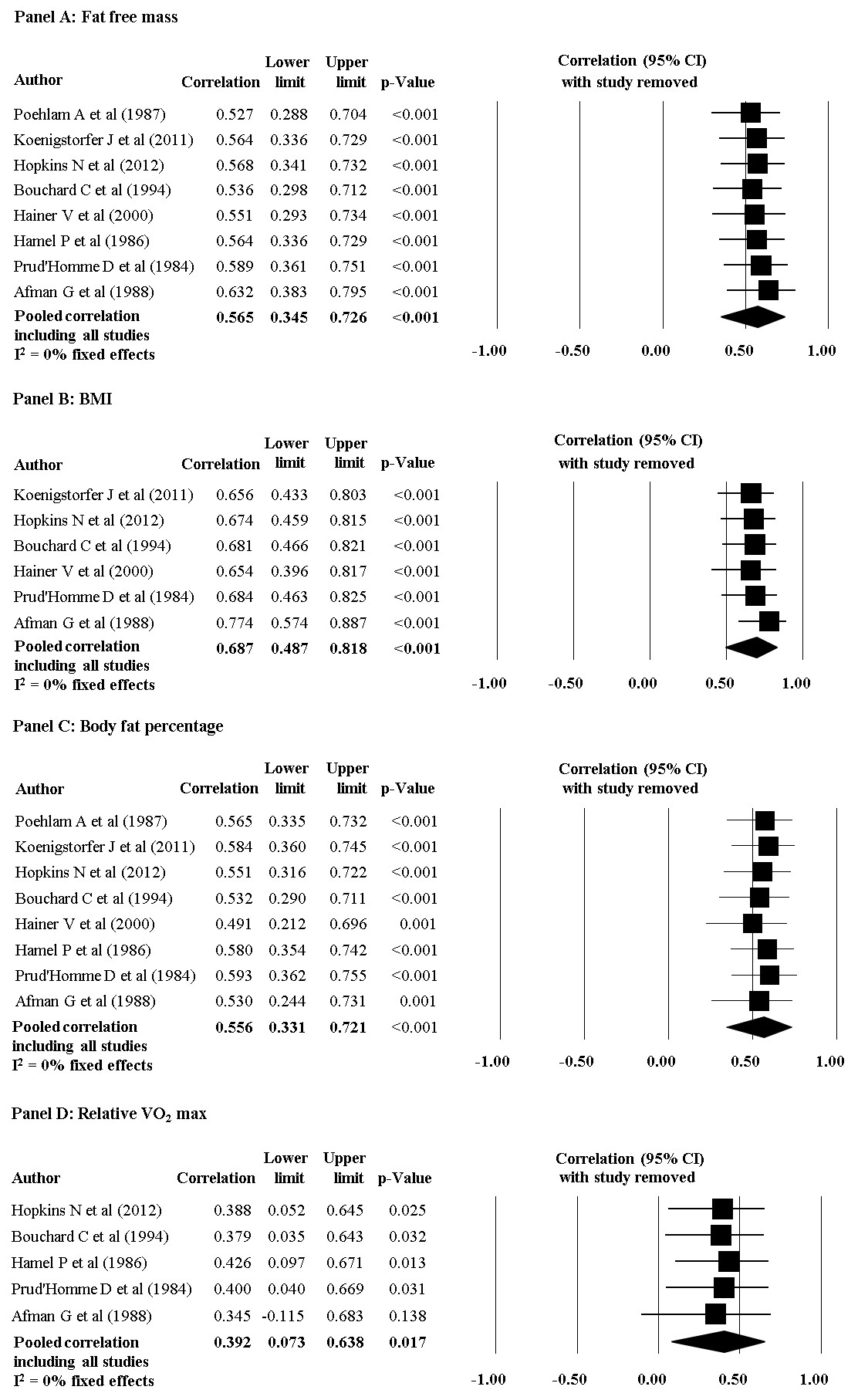

Supplement: Additional file 2: — Sensitivity analysis excluding one study at a time. (JPG 363 kb) [file 40798_2016_73_MOESM2_ESM.jpg]
